# Isorhamnetin, A Flavonol Aglycone from *Ginkgo biloba* L., Induces Neuronal Differentiation of Cultured PC12 Cells: Potentiating the Effect of Nerve Growth Factor

**DOI:** 10.1155/2012/278273

**Published:** 2012-06-17

**Authors:** Sherry L. Xu, Roy C. Y. Choi, Kevin Y. Zhu, Ka-Wing Leung, Ava J. Y. Guo, Dan Bi, Hong Xu, David T. W. Lau, Tina T. X. Dong, Karl W. K. Tsim

**Affiliations:** Division of Life Science and Center for Chinese Medicine, The Hong Kong University of Science and Technology, Clear Water Bay, Kowloon, Hong Kong

## Abstract

Flavonoids, a group of compounds mainly derived from vegetables and herbal medicines, share a chemical resemblance to estrogen, and indeed some of which have been used as estrogen substitutes. In searching for possible functions of flavonoids, the neuroprotective effect in brain could lead to novel treatment, or prevention, for neurodegenerative diseases. Here, different subclasses of flavonoids were analyzed for its inductive role in neurite outgrowth of cultured PC12 cells. Amongst the tested flavonoids, a flavonol aglycone, isorhamnetin that was isolated mainly from the leaves of *Ginkgo biloba* L. showed robust induction in the expression of neurofilament, a protein marker for neurite outgrowth, of cultured PC12 cells. Although isorhamnetin by itself did not show significant inductive effect on neurite outgrowth of cultured PC12 cells, the application of isorhamnetin potentiated the nerve growth factor- (NGF-)induced neurite outgrowth. In parallel, the expression of neurofilaments was markedly increased in the cotreatment of NGF and isorhamnetin in the cultures. The identification of these neurite-promoting flavonoids could be very useful in finding potential drugs, or food supplements, for treating various neurodegenerative diseases, including Alzheimer's disease and depression.

## 1. Introduction

Flavonoids belong to a family of polyphenolic compounds and have been considered as substitutes for estrogen [1–3].They are widely present in our daily diet and also serve as major ingredients of vegetables and herbal supplements. Chemically, flavonoid is dividing into different subclasses including flavanone, flavone, flavonol, flavanonol, isoflavone, chalcone, and others. Recently, attentions have been focused on the neurobeneficial effects of different classes of flavonoids, including neuroprotection against neurotoxin stress, promotion of memory, and learning and cognitive functions. Indeed, the protective functions of flavonoids have been reported in various bioassay systems [3–6]. Interestingly, the beneficial effects of flavonoids are not restricted to mediate the neuroprotection. Different lines of evidence indicated that flavonoids also possessed biological activities in promoting neuronal differentiation. Therefore, flavonoids could serve as one of the resources in developing new drugs, or food supplements, for the prevention of neurodegenerative diseases, for example, Alzheimer's disease and depression. Moreover, the low toxicity of flavonoids in humans has been known [1, 2].

Cultured pheochromocytoma PC12 cell line is commonly being used for the detection of neuronal differentiation in responding to various stimuli, for example, nerve growth factor (NGF) [7–9]. By measuring the length of neurite or the number of cells processing neurites, the status of differentiated PC12 cells could be determined. In addition, the neuronal differentiation could be determined biochemically in analyzing the expression of neurofilaments (NFs) that are the major structural components of the differentiated neurons [10]. Three mammalian neurofilament subunits, NF68 (*M*_*r*_ at ~68 kDa), NF160 (*M*_*r*_ at ~160 kDa), and NF200 (*M*_*r*_ at ~200 kDa), are believed to form heterodimers in making the structural domain of neurites [11].

Here, the length of neurites and the expression of neurofilaments were determined in cultured PC12 cells under the treatment of different subclasses of common flavonoids. Isorhamnetin, a flavonol aglycone from *Ginkgo biloba *L., was shown to induce the expression of neurofilaments and to potentiate the neurite-inducing activity of NGF. The identification of these neurite-promoting flavonoids could be very useful in finding potential drugs, or food supplements, for treating various neurodegenerative diseases.

## 2. Materials and Methods

### 2.1. Chemicals and Flavonoids

Isorhamnetin and other flavonoids were purchased from National Institute for the Control of Pharmaceutical Biology Products (NICPBP; Beijing, China), or Sigma (St. Louis, MO, USA) or Wakojunyaku (Osaka, Japan) or Kunming Institute of Botany, Chinese Academy of Science (Kunming, China) and solubilized in dimethylsulfoxide (DMSO) to give stock solution at a series of concentration from 25–100 mM, stored at −20°C. The MEK1/2 inhibitor U0126 was purchased from Sigma.

### 2.2. Cell Culture and Flavonoid Treatment

Pheochromocytoma PC12 cells, a cell line derived from rat adrenal medulla, were obtained from American Type Culture Collection (ATCC, Manassas, VA, USA), and which were maintained in Dulbecco's modified Eagle's medium supplemented with 6% fetal calf serum, 6% horse serum, 100 units/mL penicillin, and 100 *μ*g/mL streptomycin in a humidified CO_2_ (7.5%) incubator at 37°C. Fresh medium was supplied every other day. All culture reagents were purchased from Invitrogen Technologies (Carlsbad, CA, USA). During the treatment with flavonoids, cultured PC12 cells were serum starved for 3 hours in Dulbecco's modified Eagle's medium supplemented with 1% fetal calf serum, 1% horse serum, and penicillin-streptomycin, and then were treated with the flavonoids and/or other reagents for 72 hours. In analyzing the signaling pathway, the cells were pretreated with the MEK1/2 inhibitor U0126 (20 *μ*M) for 3 hours before the exposure to flavonoid or NGF.

### 2.3. Cell Viability Test

Cell viability was assessed by MTT [3-(4, 5-dimethyl-2-thiazolyl)-2, 5-diphenyl-2H-tetrazolium bromide] assay [12]. PC12 cells were seeded in the 96-well plate and incubated for 24 hours. After that, cells were treated with the flavonoids, or other chemicals, for another 72 hours. Then, the MTT solution was added to the cell cultures and incubated for 1 hour at 37°C. Absorbance was measured at 570 nm in a microplate reader (Thermo Scientific, Fremont, CA, USA).

### 2.4. Western Blot Analysis

After the indicated time of treatment, the cells were solubilized in lysis buffer containing 0.125 M Tris-HCl, pH 6.8, 4% SDS, 20% glycerol, 2% 2-mercaptoethanol, and analyzed immediately or stored frozen at −20°C. Proteins were separated on the 8% SDS-polyacrylamide gels and transferred to the nitrocellulose. Transfer and equal loading of the samples was confirmed by staining the Ponceau-S. The nitrocellulose was blocked with 5% fat-free milk in Tris-buffer saline/0.1% Tween 20 (TBS-T), and then incubated in the primary antibody diluted in 2.5% fat-free milk in TBS-T for 2 hours in the room temperature. The primary antibodies used were: anti-NF200 (Sigma), anti-NF160 (Sigma), anti-NF68 (Sigma), anti-glyceraldehyde 3-phosphate dehydrogenase (GAPDH; Abcam Ltd., Cambridge, UK), anti-phospho-TrkA (Cell signaling, Danvers MA, USA), anti-TrkA (Cell Signaling), anti-phospho-Akt (Cell Signaling), anti-Akt (Cell Signaling), anti-phospho-Erk1/2 (Cell Signaling), and anti-Erk1/2 (Cell Signaling). After that, the nitrocellulose was rinsed with TBS-T and incubated for 1 hour at the room temperature in peroxidase- (HRP-)conjugated anti-mouse secondary antibody (Invitrogen), or peroxidase- (HRP-)conjugated anti-rabbit secondary antibody (Invitrogen), diluted in the 2.5% fat-free milk in TBS-T. After intensive washing with TBS-T, the immune complexes were visualized using the enhanced chemiluminescence (ECL) method (GE Healthcare, Piscataway, NJ, USA). The intensities of the bands in the control and different samples, run on the same gel and under strictly standardized ECL conditions, were compared on an image analyzer, using a calibration plot constructed from a parallel gel with serial dilutions of one of the samples.

### 2.5. Neurite Outgrowth Assay

Cultured PC12 cells were treated with isorhamnetin and/or NGF for 72 hours, with fresh medium and reagents supplied every 24 hours. A light microscope (Diagnostic Instruments, Sterling Heights, MI, USA) equipped with a phase-contrast condenser, 10x objective lens and a digital camera (Diagnostic Instruments) were used to capture the images with the manual setting. For analyzing the number and length of neurite, approximately 100 cells were counted from at least 10 randomly chosen visual fields for each culture. Using the photoshop software, the cells were then analyzed for the number and length of neurite. The cells were scored as differentiated if one or more neurites were longer than the diameter of cell body, and they were also classified to different groups according to the length of neurite that it possessed, that is, <15 *μ*m, 15–30 *μ*m, and >30 *μ*m.

### 2.6. Statistical Analysis and Other Assays

Statistical analyses were performed using one way ANOVA followed by the Students *t*-test. Statistically significant changes were classed as * where *P* < 0.05; ** where *P* < 0.01; *** where *P* < 0.001.

## 3. Results

### 3.1. Effect of Flavonoids on the Differentiation of PC12 Cells

**Figure 1:**
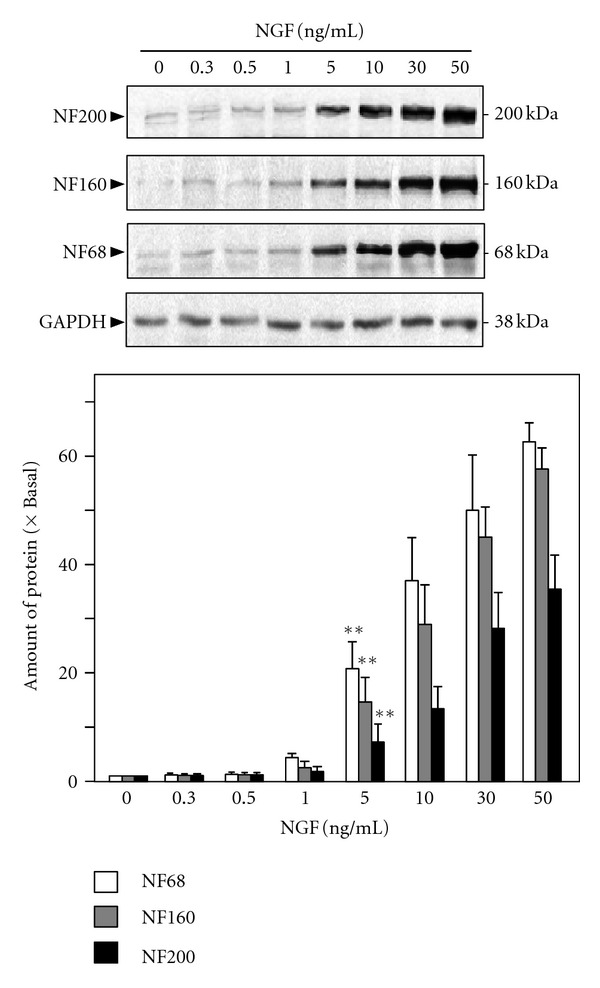
NGF induces the expression of neurofilaments in cultured PC12 cells. Cultured PC12 cells were treated with NGF (0.3 to 50 ng/mL) for 72 hours. The cell lysates were collected to determine the expressions of NF68 (*M*_*r*_ ~ 68 kDa), NF160 (*M*_*r*_ ~ 160 kDa), and NF200 (*M*_*r*_ ~ 200 kDa). GADPH (*M*_*r*_ ~ 38 kDa) served as a loading control (upper panel). Quantification plot was shown in lower panel. Values are expressed as the fold of change (×Basal) against the control (no treatment; set as 1), and in Mean ± SEM, *n* = 4, each with triplicate samples. Representative images were shown. **P < 0.01 compared to the control.

Sixty-five flavonoids from different subclasses were screened for their differentiating effect on cultured PC12 cells. These flavonoids are mainly derived from health foods and Chinese herbal medicines. To enhance the efficiency of the screening platform, the first screening test was done on the expression of neurofilaments, including NF68, NF160, and NF200, instead of the extension of neurite. Indeed, application of NGF in cultured PC12 cells induced the expression of neurofilaments in a dose-dependent manner (Figure 1). Up to 5 ng/mL of NGF, the increased expressions of NF68 (at ~68 kDa) and NF160 (at ~160 kDa), and NF200 (at ~200 kDa) that could be significant are revealed here. The NGF-induced expression was more robust for NF68 and NF160 induction: the maximal expression at 50 ng/mL NGF was ~50 folds. The maximal induction of NF200 was over 30 folds.

To screen the potential neuronal differentiation effect of flavonoids, different flavonoids were applied onto cultured PC12 cells for 72 hours in different concentrations: these concentrations (e.g., isorhamnetin) had neither cytotoxicity nor proliferating effect, as achieved from the MTT assay (Supplementary figure available online at doi: 10.1155/2012/278273). After the treatment, the cells were collected to perform western blot analysis to determine the expression levels of NF68, NF160, and NF200. Some of the flavonoids increased the expression levels of neurofilaments (Table 1). For those having strong inductive effects (i.e., > ±±) were: hesperidin from *Citrus medica *var.* sarcodactylis* and *Citrus limonum* var.* dulcis*, luteolin from *Flos lonicerae*, sulphuretin from *Cotinus* family, daidzein, genistein and glycitein from *Glycine max *(L.) Merr., tectoridin from *Belamcanda chinensis*, cardamonin from *Alpinia katsumadai*, and kaempferol, quercetin, and isorhamnetin from *G. biloba.* Among these flavonoids, the flavonol aglycone, isorhamnetin, was found to have the most evident effect in inducing the expression of neurofilaments in PC12 cells. Thus, isorhamnetin was chosen for further investigation.

**Figure 2:**
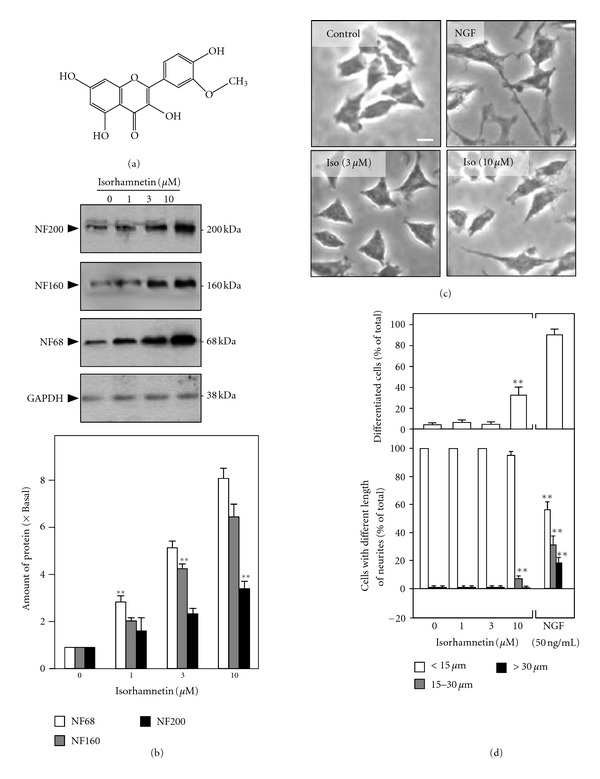
Isorhamnetin induces the neurofilament expression in cultured PC12 cells but not the neurite outgrowth. (a) The chemical structure of isorhamnetin is illustrated. (b) Cultured PC12 cells were treated with isorhamnetin (1 to10 *μ*M) for 72 hours. The cell lysates were collected to determine the expressions of NF68, NF160, and NF200 (upper panel). GADPH served as a loading control. The lower panel shows the quantitation from the blots by a densitometer. Values are expressed as the fold of change (× Basal) against the control (no treatment; set as 1), and in mean ± SEM, *n* = 4, each with triplicate samples. (c) Cultures were treated with isorhamnetin (3 or 10 *μ*M) and NGF (50 ng/mL), as indicated, for 72 hours. Cells were fixed with ice-cold 4% paraformaldehyde. Bar = 10 *μ*m. Representative images were shown. (d) Cultured PC12 cell was treated as in (c). The % of differentiated cell (upper panel) and length of neurite (lower panel) were counted as described in the Materials and Methods section. Values are expressed as % of total cells in 100 counted cells, mean ± SEM, *n* = 4. **P < 0.01 compared to the control.

Isorhamnetin is a flavonol aglycone (Figure 2(a)). In the cultures treated with isorhamnetin, the expressions of NF68, NF168, and NF200 were increased: the neurofilament induction was in a dose-dependent manner (Figure 2(b)). The protein induction was significantly revealed at 1 *μ*M of isorhamnetin. Under 10 *μ*M of isorhamnetin in the cultures, the expressions of NF68 and NF160 were increased over 6 folds: while NF200 was significantly altered to over 3 folds. The expression level of control protein GAPDH was unchanged (Figure 2(b)). The outgrowth of neurite was subsequently analyzed in isorhamnetin-treated PC12 cells. The effect of isorhamnetin in inducing neurite outgrowth of cultured PC12 cells was not significant (Figure 2(c)), at lease under the low concentration at 3 *μ*M. At higher concentration of isorhamnetin (10 *μ*M), the differentiated cell was revealed, but the induction was much less than that of NGF at 50 ng/mL (Figure 2(c)). Quantitation was performed on the extent of those neurites. Counting the number of differentiated cells (i.e., having a neurite longer than the cell body), only ~30% of total cell population could be considered as differentiated under the treatment of 10 *μ*M isorhamnetin (Figure 2(d), *upper panel*). Low concentration of isorhamnetin did not show any induction effect. In contrast, NGF at 50 ng/mL induced the cell differentiation almost to 100%. The length of neurite was also measured: the number of cells possessing neurite length at 15–30 *μ*m was significantly increased (~10%) in 10 *μ*M isorhamnetin-treated cells (Figure 2(d), *lower panel*). Thus, the neurite-inducing effect of isorhamnetin in cultured PC12 cells was very little as compared to that of NGF at 50 ng/mL.

### 3.2. Isorhamnetin Potentiates the NGF-Induced Differentiation of PC12 Cells

**Figure 3:**
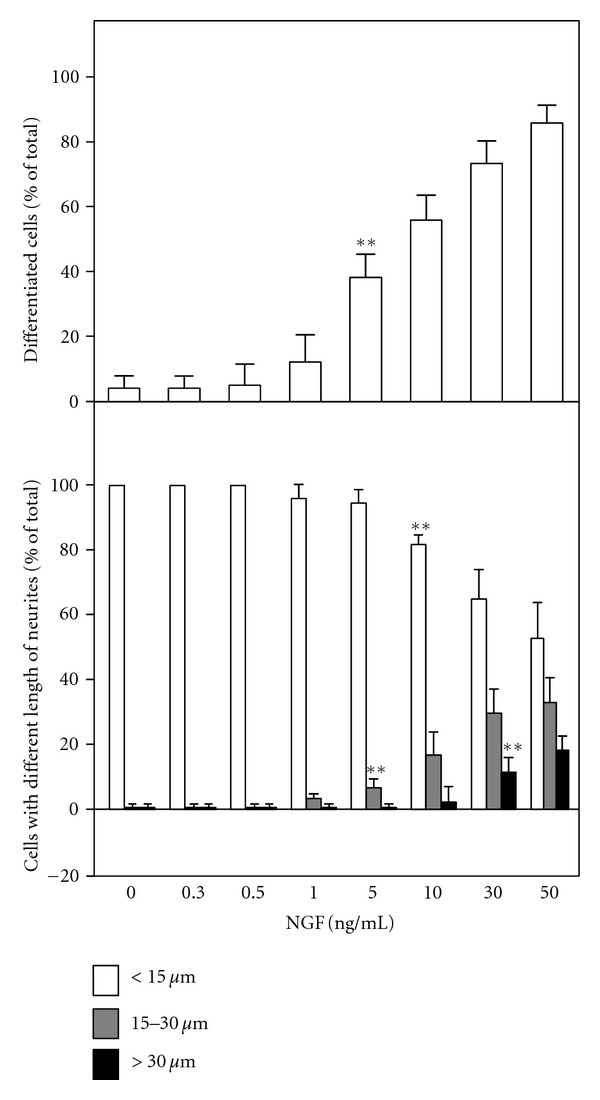
NGF induces the neurite outgrowth in a dose-dependent manner. Cultured PC12 cultures were treated with NGF (0.3 to 50 ng/mL) for 72 hours. Cells were fixed with ice-cold 4% paraformaldehyde. The % of differentiated cell (upper panel) and length of neurite (lower panel) were counted as described in the Method section. Values are expressed as % of total cells in 100 counted cells, Mean ± SEM, *n* = 4. **P < 0.01 compared to the control.

Since isorhamnetin did not seem to have a significant effect on neurite outgrowth of PC12 cells, we therefore aimed to search for the collaborative effect of this flavonoid when applied together with NGF. First, a suitable concentration of NGF was selected: this concentration should have no effect on the neurite outgrowth and/or the neurofilament expression. The concentration of NGF below 1 ng/mL did not show any significant effect on the number of differentiated cell and/or the neurite outgrowth of cultured PC12 cells (Figure 3). Moreover, the expression of neurofilament was not increased under NGF concentration below 1 ng/mL (see Figure 1). Under this scenario, 0.5 ng/mL, a concentration of NGF at which it showed no induction effect at all, was used here to cotreat PC12 cultures together with isorhamnetin.

**Figure 4:**
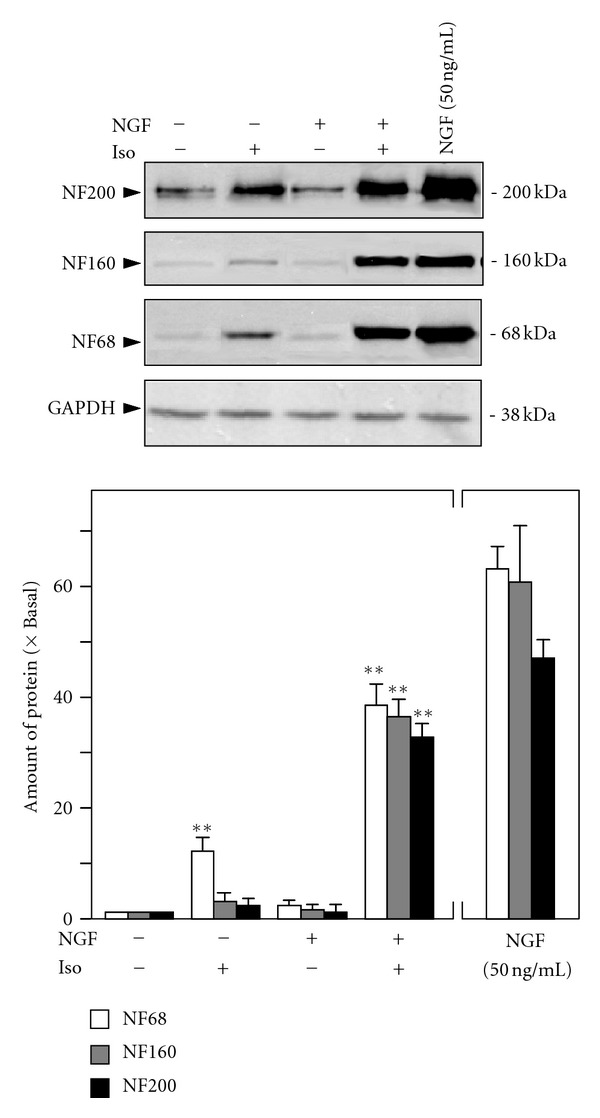
Isorhamnetin potentiates the NGF-induced neurofilament expression. Cultured PC12 cells were treated with NGF (0.5 ng/mL), isorhamnetin (10 *μ*M), and NGF (0.5 ng/mL) + isorhamnetin (10 *μ*M) for 72 hours. NGF at 50 ng/mL was applied as a control. The cell lysates were collected to determine the expressions of NF68, NF160, and NF200 (upper panel). GADPH served as a loading control. Quantification plot was shown in lower panel. Values are expressed as the fold of change (× Basal) against the control (no treatment; set as 1), and in mean ± SEM, *n* = 4. Representative images were shown. ** where *P* < 0.01 compared to the control.

**Figure 5:**
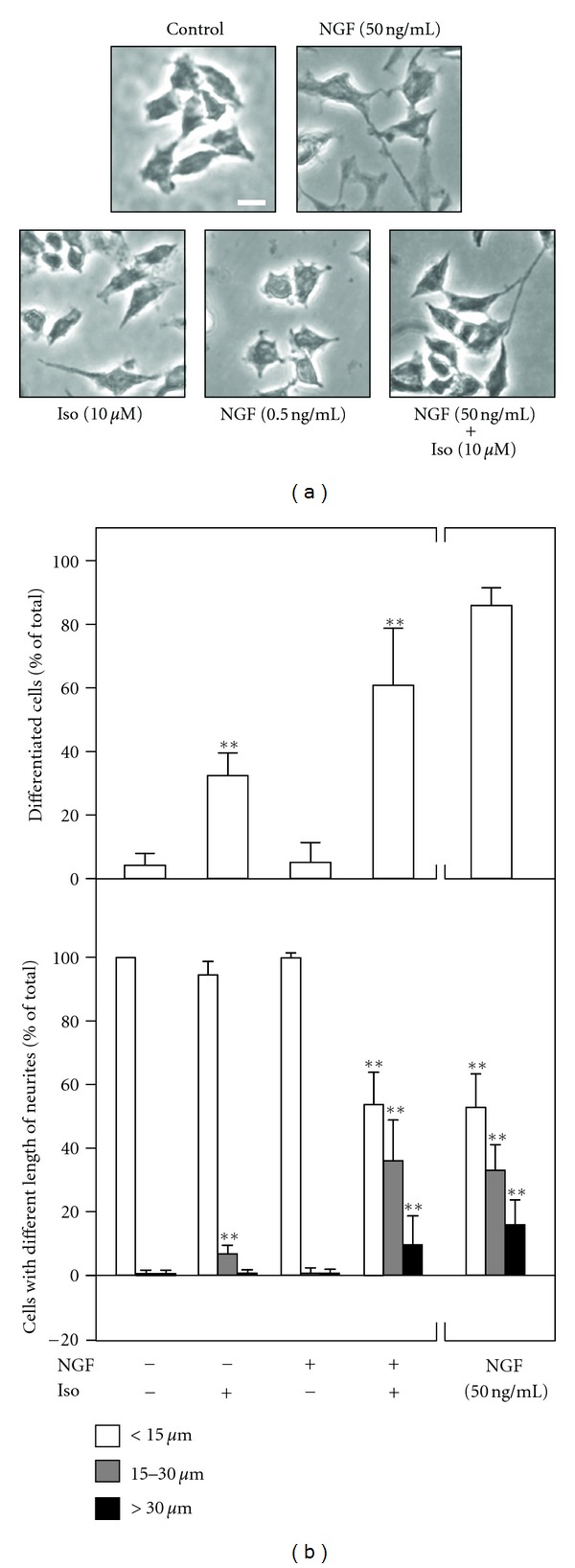
Isorhamnetin potentiates the NGF-induced neurite outgrowth. Cultured PC12 cells were treated with NGF (0.5 ng/mL), isorhamnetin (10 *μ*M), and NGF (0.5 ng/mL) + isorhamnetin (10 *μ*M) for 72 hours, as in Figure 4. (a) Cells were fixed with ice-cold 4% paraformaldehyde and the extension of neurites was revealed. Bar = 10 *μ*m. (b) The % of differentiated cell (upper panel) and length of neurite (lower panel) were counted as described in the Method section. Values are expressed as % of cells in 100 counted cells, mean ± SEM, *n* = 4. **P < 0.01 compared to the control.

Isorhamnetin (10 *μ*M) and NGF (0.5 ng/mL) were coapplied in cultured PC12 cells for 72 hours. Then, the cultures were collected to perform Western blot analysis to determine the change of neurofilament expression, including NF68, NF160, and NF200. NGF at 0.5 ng/mL showed no effect on neurofilament expression, while isorhamnetin at 10 *μ*M only showed the induction of NF68 at ~10% (Figure 4). The cotreatment of isorhamnetin and NGF robustly increased the expressions of neurofilaments, that is, NF68, NF160 and NF200. The induction of these neurofilaments was over 30 folds: this magnitude of induction shared a similarity to that of high concentration of NGF at 50 ng/mL (Figure 4). In addition, the outgrowth of neurite in the cultures was analyzed. The cotreatment of isorhamnetin (10 *μ*M) and NGF (0.5 ng/mL) induced the differentiation of cultured PC12 cells, and the outgrowth of neurite was clearly revealed in the cotreatment (Figure 5(a)). In addition, the number of differentiated cells significantly increased by ~60% after this cotreatment (Figure 5(b), *upper panel*), and which also induced the length of neurite (Figure 5(b),* lower panel*). After the cotreatment, those cells having the long neurite, for example, above 15 *μ*m in length, were markedly increased: this induction effect was similar to that of high concentration of NGF at 50 ng/mL (Figure 5(b), *lower panel*). These results therefore suggested the potentiating role of isorhamnetin in the neurite outgrowth activity of NGF.

### 3.3. The Effect of Isorhamnetin on NGF-Induced Signaling Pathways

**Figure 6:**
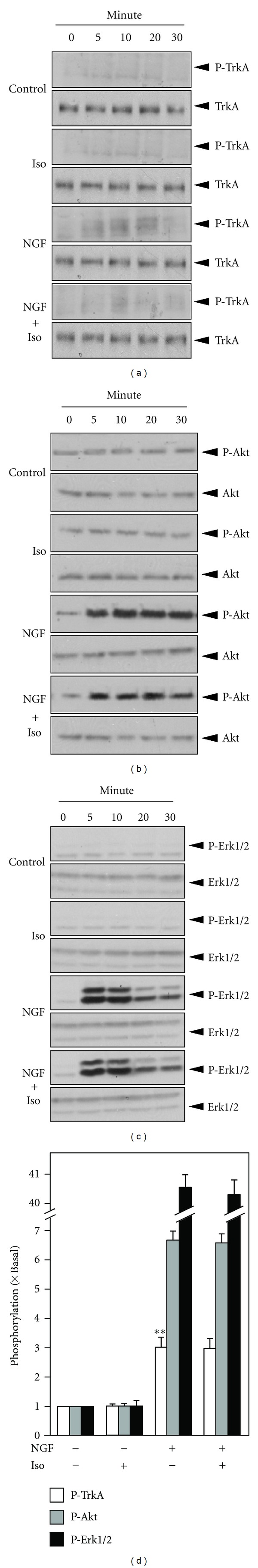
The potentiating effect of isorhamnetin is not mediated by NGF-induced signaling cascade. Cultured PC12 cells, serum starvation for 5 hours, were treated with NGF (5 ng/mL), isorhamnetin (Iso; 10 *μ*M), and NGF (5 ng/mL) + isorhamnetin (Iso; 10 *μ*M) for different time. Total TrkA and phosphorylated TrkA (a) total Akt and phosphorylated Akt (b) total Erk1/2 and phosphorylated Erk1/2 (c) were revealed by using specific antibodies. (d) Quantification plot of the phosphorylation level in treatment of 5 min was shown. Values are expressed as the fold of change (×Basal) against the control (no treatment; set as 1), and in mean ± SEM, *n* = 4. Representative images were shown. ** where *P* < 0.01 compared to the control.

**Figure 7:**
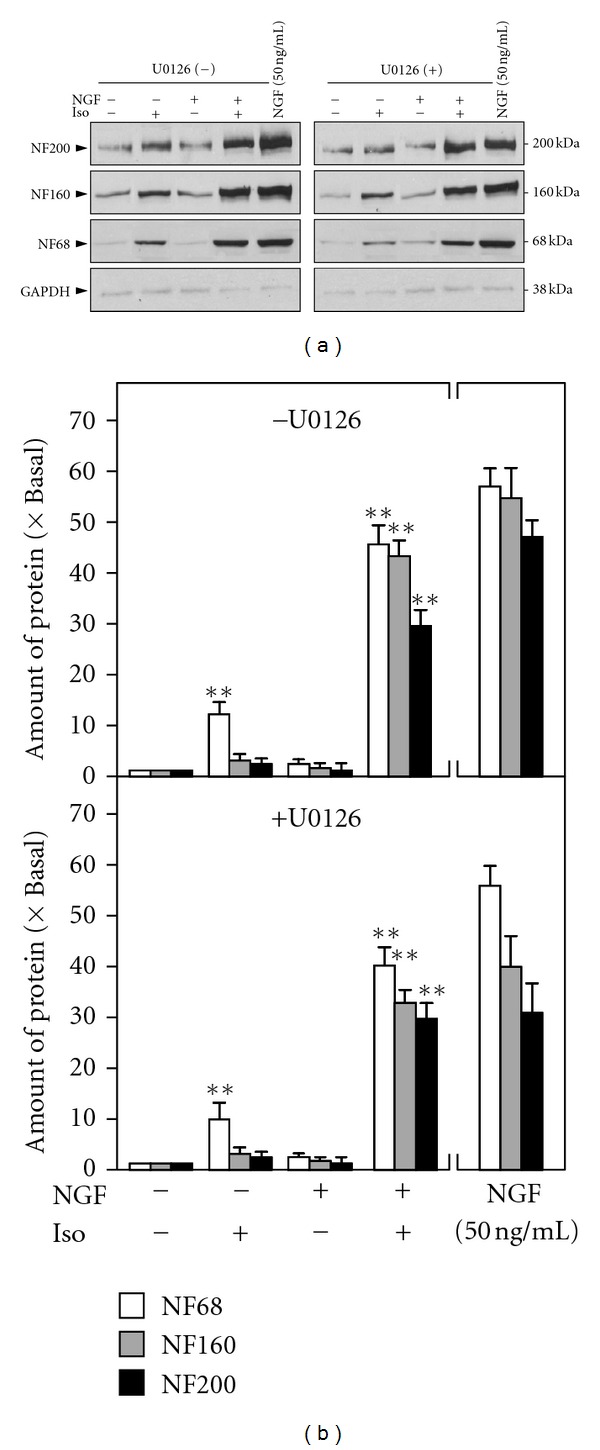
The potentiating effect of isorhamnetin on NGF-induced response could not be blocked by U0126. Cultured PC12 cells, serum starvation for 5 hours, were treated with NGF (0.5 ng/mL), isorhamnetin (Iso; 10 *μ*M), and NGF (0.5 ng/mL) + isorhamnetin (Iso; 10 *μ*M) for 72 hours with or without the pretreatment of U0126 (20 *μ*M) for 3 hours. NGF at 50 ng/mL served as a positive control. (a) The cell lysates were collected to determine the expressions of NF68, NF160, and NF200. GADPH served as a loading control. (b) Quantification plot was shown in lower panel. Values are expressed as the fold of change (×Basal) against the control (no treatment; set as 1), and in mean ± SEM, *n* = 4. Representative images were shown. **where *P* < 0.01 compared to the control.

To explore the mechanism of isorhamnetin-induced neurofilament expression and its potentiating effect on NGF-induced neurite outgrowth, the effects of isorhamnetin in phosphorylating the NGF-induced signaling molecules was tested. NGF, or isorhamnetin, was applied onto the serum-starved PC12 cell cultures. After treatment at different time periods, the cell lysates were collected to perform western blotting, as to reveal the phosphorylation levels of various signaling molecules. NGF at low level induced the phosphorylations of TrkA (~140 kDA), Erk1/2 (~44/42 kDa), and Akt (~60 kDa) within 5 min (Figure 6). However, isorhamnetin could not induce the phosphorylation of any of these molecules, even up to 30 min of treatment. To test the possible potentiating effect of isorhamnetin in the NGF-activated signaling, we cotreated isorhamnetin with NGF at 5 ng/mL. Here, NGF in 5 ng/mL was the lowest concentration to phosphorylate the molecules (Figure 6); however, the cotreatment with isorhamnetin did not enhance the phosphorylation. To further confirm the role of MEK pathway in the function of isorhamnetin, the pretreatment of U0126 at 20 *μ*M was applied onto the cultures, as to block the mitogen-activated protein kinase signaling. Results showed that U0126 did not block the neurofilament expression induced by isorhamnetin, or the potentiating effect of isorhamnetin, on NGF-induced neurite outgrowth (Figure 7). In contrast, this concentration of U0126 was demonstrated to partially blocked the NGF signaling (data not shown here), and which was in line to previous studies [13, 14]. These results suggested that the response triggered by isorhamnetin could be very different to that of NGF in the cultures.

## 4. Discussion

Sixty-five flavonoids were screened for their differentiating effect on cultured PC12 cells. Over 20 of them showed inductive effect on the expression of neurofilaments; however, which did not simultaneously induce the neurite outgrowth in the cultures. Isorhamnetin, a flavonol aglycone isolated mainly from *G*.  *biloba*, was chosen to do the following studies including the cotreatment with NGF in low concentration. However, the effect of isorhamnetin on neurite outgrowth was very limited. Thus, the expression of neurofilament and neurite outgrowth in cultured PC12 cells could be two independent events. On the other hand, isorhamnetin showed a robust effect in potentiating the neurite-inducing activity of NGF, that is, the coapplication of isorhamnetin with low concentration of NGF (0.5 ng/mL) could achieve the effect as that of high concentration of NGF (50 ng/mL). Therefore, the NGF-potentiating effect of isorhamnetin could be considered as a new direction in developing health food supplements to help the recovery of neurodegenerative diseases relating to NGF insufficient.

Ginkgo leaf extract is the most popular herbal supplement being sold in Europe and the USA, where it is used to treat the symptoms of early-stage Alzheimer's disease, vascular dementia and tinnitus of vascular origin [15]. The most well-known standardized preparation of Ginkgo extract on the current herbal market is Egb 761 that consists of two major groups of substances: the flavone glycosides (flavonoid fraction, 24%) and the terpene lactones (terpenoid fraction, 6%) [16]. The amount of isorhamnetin in dry Ginkgo leaf could reach 0.85 ± 0.02 mg/g [17]. Isorhamnetin has been proved to have the activities of antitumor [18], anti-oxidation [3, 19], reducing the superoxide anion in liver cells [20], and decreasing the risk of many disorders, for example, diabetes, hypertension, and heart disease [21]. In nervous system, isorhamnetin was also shown to have the protective effect against the oxidative stress induced by simulated microgravity *in vitro* [22]. Besides isorhamnetin, other flavonoids, or natural compounds, have also been shown to induce neuronal differentiation and neurite outgrowth, for example, wogonin isolated from *Scutellaria baicalensis* [23] and euxanthone isolated from *Polygala caudate *[24]. In addition, the NGF-potentiating flavonoids have also been reported, for example, liquiritin from *Glycyrrhizae *root [25] and littorachalcone from *Verbena litoralis* [26].

Neurofilaments are the key components during the extension of neurite, and their expression level could serve as a marker for neuronal differentiation. The application of isorhamnetin in cultured PC12 cells could increase significantly the expression levels of NF68 and NF160. Both NF68 and NF160 are the protein markers for the early stage of the differentiation. In contrast, the expression of NF200, a marker protein for late stage of neuron differentiation [27], was also altered but at a less extent as compared to that of NF68 or NF160 at the isorhamnetin-treated cultures. Under this scenario, the involvement of isorhamnetin in neuronal differentiation could be mainly at the early stage, which however could not fully support the entire differentiation process at late stage. The potentiating effect of isorhamnetin in the NGF-induced neurite outgrowth also supported this notion. The increased expressions of NF68 and NF160 in cultured cells, induced by isorhamnetin, could be a prelude for the expression of NF200, and the neurite outgrowth could be the final outcome of increased protein expressions.

NGF is one of the key modulators of neurite outgrowth during development and into adulthood, many diseases of nervous system are associated with NGF insufficiency, especially some neurodegenerative diseases [28], for example, depression [29] and Alzheimer's disease [30]. For the property of potentiating effect on NGF-induced neurite outgrowth, isorhamnetin would have potential to be used to treat the differentiation problem caused by NGF insufficiency. Therefore, the NGF-potentiating effect of isorhamnetin could be considered as a new direction in developing drugs or health food supplements to help the prevention and recovery of neurodegenerative diseases. NGF achieves its function by binding and activating TrkA receptor on neuronal cells. The NGF-activated TrkA stimulates downstream signaling pathways, which results in neuronal differentiation and promoting cell survival [13]. The NGF-induced neurite outgrowth is mediated by activation of Ras/ERK, PI3K/Akt, and phospholipase-C-*γ* (PLC-*γ*1) [14]. Various classes of flavonoids were demonstrated to induce the neurite outgrowth, or to potentiate the NGF-induced neurite outgrowth, in cultured neurons, and the signal could be mediated by a MEK pathway [31–33]. Here, isorhamnetin can neither directly activate these signaling molecules by phosphorylation, nor potentiated NGF-induced activation of the signaling pathways, which may tell us that even though different flavonoids have the similar effects in neurite outgrowth, their mechanism may be totally different. The signal triggered by isorhamnetin in cultured PC12 cells is being determined currently in our laboratory.

## 5. Conclusion

 Flavonoids are a group of natural compounds with multiple biofunctions. In this study, we aimed to investigate their effects on neuronal differentiation of cultured PC12 cells. Sixty-five flavonoids from different subclasses were screened for their differentiating effect on cultured PC12 cells. Among these flavonoids, a flavonol aglycone, isorhamnetin was found to have the best effect in inducing the expression of neurofilaments, and which potentiated the NGF-induced neurite outgrowth and neurofilament expression. Although the mechanism has not been revealed, this property of isorhamnetin could be a new direction in searching potential candidates as new drugs or food supplements for neurodegenerative diseases.

## Supplementary Material

Supplementary figure: The cytotoxicity of isorhamnetin in PC12 cells PC12 cells were seeded on to 96-well plate and incubated for 24 hours. After that, the cells were treated with isorhamnetin in different concentration for another 72 hours. The MTT solution was added to the cell cultures and incubated for 1 hour at 37 °C. Absorbance was measured at 570 nm in a microplate reader. Values are expressed as the % of total cell number against the control (0.02% DMSO), and in Mean ± SEM, n=4.

## Figures and Tables

**Table 1:** Flavonoids induce the expressions of NF68, NF160, and NF200.

Flavonoid	NF68	NF160	NF200	Flavonoid	NF68	NF160	NF200	Flavonoid	NF68	NF160	NF200
*Flavanones*				*Biflavones*				*Chalcones*			
Alpinetin	−	−	−	Ginkgetin	+	+	−	Cardamonin	++	+	+
Farrerol	−	−	−	*Dihydrochalcones*				*Flavanes*			
Hesperidin	++	+	−	Phloretin	−	−	−	(−)-Catechin	−	−	−
Liquiritin	−	−	−	Phloridzin	−	−	−	(−)-Epicatechin	−	−	−
Naringenine	+	−	−	*Flavanonols*				*Flavonols*			
Naringin	−	−	−	Dihydromyricetin	−	−	−	Astragalin	−	−	−
Neohesperidin	−	−	−	Silybin	−	−	−	Galangin	+	+	−
Prunin	−	−	−	*Isoflavones*				Hibifolin	−	−	−
*Flavones*				Calycosin	−	−	−	Hyperin	+	+	−
Apigenin	−	−	−	Calycosin-7-O-glc	−	−	−	Icariin	−	−	−
Apiin	−	−	−	Daidzein	++	+	−	Isoquercitrin	−	−	−
Baicalein	−	−	−	Daidzin	−	−	−	Isorhamnetin	+++	+++	+
Baicalin	−	−	−	Formononetin	−	−	−	Isorhamnetin-3-O-rut	−	−	−
Chrysin	−	−	−	Genistein	++	+	−	Kaempferol	++	+	+
Hebacetin-8-OCH_3_	+	−	−	Genistin	−	−	−	Kaempferol-3-O-rut	−	−	−
Isovitexin	−	−	−	Glycitein	++	+	−	Kaempferol-3-O-glc	+	−	−
Luteolin	++	+	+	Glycitin	−	−	−	Quercetin	++	+	−
Lysionotin	−	−	−	Irisflorentin	+	+	−	Quercetin-3^′^-O-glc	+	−	−
Morusin	−	−	−	Ononin	−	−	−	RNFG	+	+	−
Scutellarin	−	−	−	Pratensein	+	+	−	Rutin	−	−	−
Scoparin	+	−	−	Puerarin	−	−	−	Tiliroside	−	−	−
Tangeretin	+	−	−	4^′^,7-OCH_3_-puerarin	−	−	−	Vitexicarpin	−	−	−
Wogonin	−	−	−	4^′^,7-OCOCH_3_-puerarin	−	−	−				
*Aurones*				Tectoridin	++	+	−				
Sulphuretin	++	+	−	Tectorigenin	−	−	−	NGF	+++	+++	+++

Data are means ± SEM, *n* = 3. Data are based on the means. And the value of SEM is within 5% of the mean, which is not shown for clarity. “+” to “+++” indicate the percentage of increasing of the neurofilaments expression level (“+” indicates 100% to 200% increasing in the tested activities, “++” indicates 200% to 300% increasing, and “+++” indicates >300% increasing). “−” indicates no effect, that is, below 10%. For the tested flavonoids, the maximal testing concentrations distribute from 3 *μ*M to 30 *μ*M, according to the results from cell viability assay. The submaximal doses of these flavonoids were used for comparison. NGF at 50 ng/ml served as a positive control. RNFG is corresponding to Radix Notoginseng flavonol glucoside or quercetin 3-O-*β*-D-xylopyranosyl-*β*-D-galactopyranoside.
