# Supplementary material for: Isorhamnetin, A Flavonol Aglycone from Ginkgo biloba L., Induces Neuronal Differentiation of Cultured PC12 Cells: Potentiating the Effect of Nerve Growth Factor
Source: Evid Based Complement Alternat Med. 2012 Jun 17;2012:278273. doi: 10.1155/2012/278273 (PMC3385709; doi:10.1155/2012/278273)
Supplement: Supplementary file 1 — Supplementary figure: The cytotoxicity of isorhamnetin in PC12 cells PC12 cells were seeded on to 96-well plate and incubated for 24 hours. After that, the cells were treated with isorhamnetin in different concentration for another 72 hours. The MTT solution was added to the cell cultures and incubated for 1 hour at 37 °C. Absorbance was measured at 570 nm in a microplate reader. Values are expressed as the % of total cell number against the control (0.02% DMSO), and in Mean ± SEM, n=4. [file 278273.f1.pdf]

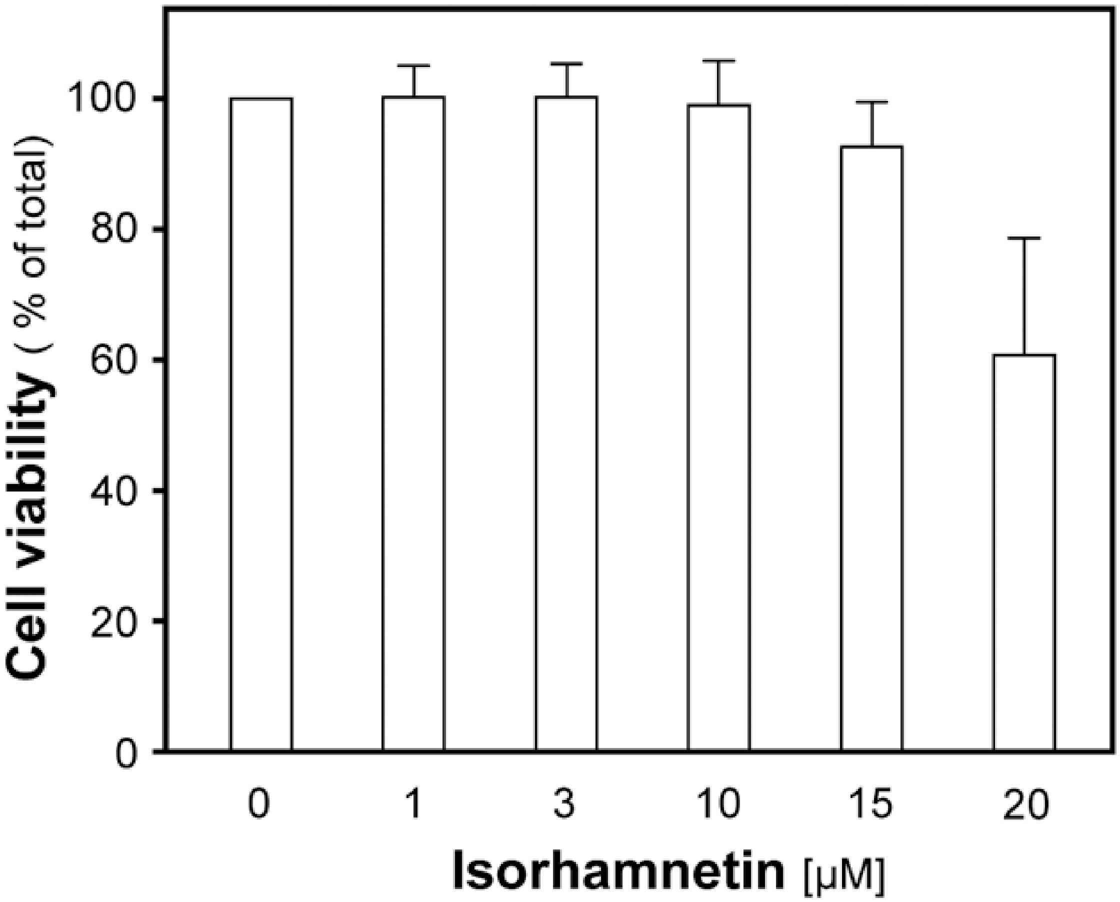

Supplementary figure: The cytotoxicity of isorhamnetin in PC12 cells

PC12 cells were seeded on to 96-well plate and incubated for 24 hours. After that, the cells were treated with isorhamnetin in different concentration for another 72 hours. The MTT solution was added to the cell cultures and incubated for 1 hour at 37 ^{o}C. Absorbance was measured at 570 nm in a microplate reader. Values are expressed as the % of total cell number against the control (0.02% DMSO), and in Mean $\pm$ SEM, *n*=4.
